# COVID-19 pandemic moral injury in healthcare professionals: a systematic review

**DOI:** 10.1192/bjo.2021.204

**Published:** 2021-06-18

**Authors:** Verity Williams, Rhian Bradley, Rafey Faruqui, Julia Hynes, Julie Anderson

**Affiliations:** 1 Kent and Medway NHS and Social Care Partnership Trust, Kent and Medway Medical School; 2 Kent and Medway Medical School; 3 University of Kent

## Abstract

**Aims:**

Moral injury (MI) refers to psychological distress resulting from witnessing or participating in events which violate an individual's moral code. Originating from military experiences, the phenomenon also has relevance for healthcare professionals dealing with wars, natural disasters and infectious diseases. The deontological basis of medicine prioritises duty to the individual patient over duty to wider society. These values may place healthcare professionals at increased risk of moral injury, particularly in crisis contexts where they may be party to decisions to withdraw or divert care based on resource availability.

We conducted a systematic review of medical literature to understand the extent and clinical and socio-demographic correlates of moral injury during the COVID-19 pandemic.

**Method:**

We conducted a systematic review of reports included in MEDLINE, PsycINFO, BNI, CINAHL, EMBASE, EMCARE and HMIC databases using search terms: “moral injury” AND “covid” OR “coronavirus” OR “pandemic”. We also searched Google Scholar and Ovid Database and conducted reference searching. We searched for published quantitative primary research as well as advance online publications and pre-print research. Findings are reported in line with Preferred Reporting Items for Systematic Reviews and MetaAnalyses (PRISMA). Two authors independently assessed the included studies’ methodological quality using a seven-item checklist.

**Result:**

Our databases search identified 498 records and other sources identified 4 records. We screened 391 records after removing duplicates. 4 reports met our protocol requirements.

Three papers used cross-sectional designs. One reported longitudinal outcomes of their sample already described in one of the three papers. Only one study used a MI scoring system validated for healthcare professionals. Others used scoring validated in military populations. These papers reported outcomes from 3334 subjects, with a higher proportion of females. The largest study (3006 subjects) reported MI in 41.3% of their sample. Overall, factors associated with greater MI included: providing direct care to COVID-19 patients; sleep troubles; being unmarried; aged <30 years; female gender; and Buddhist/Taoist faith. Nurses reported a greater severity of MI than physicians. MI significantly correlated with anxiety, depression and burnout. The longitudinal study reported that more stressful and less supportive work environments predicted greater MI at 3 months follow-up.

The average quality assessment score of these studies was 4/7.

**Conclusion:**

It is important that we are able to address moral injury awareness training as part of workforce preparedness and burnout prevention during the COVID-19 pandemic and other disaster responses across the globe.

